# Maternal lipid profile during early pregnancy and birth weight: A retrospective study

**DOI:** 10.3389/fendo.2022.951871

**Published:** 2022-09-15

**Authors:** Si-Meng Zhu, Han-Qiu Zhang, Cheng Li, Chen Zhang, Jia-Le Yu, Yan-Ting Wu, He-Feng Huang

**Affiliations:** ^1^International Peace Maternity and Child Health Hospital, Shanghai Jiao Tong University School of Medicine, Shanghai, China; ^2^Shanghai Key Laboratory of Embryo Original Disease, Shanghai, China; ^3^Research Units of Embryo Original Diseases, Chinese Academy of Medical Sciences, Shanghai, China; ^4^Obstetrics and Gynecology Hospital, Institute of Reproduction and Development, Fudan University, Shanghai, China

**Keywords:** maternal lipid profiles, body mass index, birth weight, large for gestational age, macrosomia

## Abstract

**Introduction:**

Elevated maternal serum lipid concentrations have been related to an adverse intrauterine environment and lead to abnormal birth weight.

**Objective:**

In this study, we aimed to explore the association between maternal lipid profiles during early pregnancy and birth weight with stratified pre-pregnancy body mass index (BMI).

**Methods:**

This retrospective cohort study was based on a large population from two major maternity centers in Shanghai, China. We included 57,516 women with singleton live birth between January 2018 and October 2020. All of the enrolled women had fasting lipid concentrations measured in early pregnancy. The primary outcomes were birth weight and risks of adverse birth outcomes, including macrosomia, large for gestational age (LGA), low birth weight (LBW), and small for gestational age (SGA).

**Results:**

Higher maternal concentrations of total cholesterol (TC), triglyceride (TG), and low-density cholesterol (LDL-c) in early pregnancy were associated with increased birth weight. Ln transformed TG and levels exhibited a positive association with LGA and macrosomia (OR = 1.33, 95% CI: 1.25, 1.42 and OR = 1.37, 95% CI: 1.24, 1.52) and showed a negative relationship with SGA (OR = 0.73, 95% CI: 0.62, 0.85). High TG (>75^th^ percentile, 1.67 mmol/L) group also showed higher risks of LGA and macrosomia (OR = 1.21, 95% CI: 1.15, 1.28 and OR = 1.20, 95% CI: 1.10, 1.31) and decreased prevalence of SGA (OR = 0.71, 95% CI: 0.61, 0.83). Moreover, significant combined effects of pre-pregnancy BMI and lipid profiles on LGA and macrosomia were identified.

**Conclusions:**

Elevated maternal lipid profiles in early pregnancy are associated with higher birth weight and increased risks of LGA and macrosomia. We propose that serum lipid profiles in early pregnancy and pre-pregnancy BMI could serve as screening indexes for high-risk women.

## Introduction

According to Developmental Origins of Health and Disease (DOHAD) theory, maternal metabolism and intrauterine environment could affect fetal development and further impact their health status in adulthood (1, 2). Among the prenatal metabolic factors, maternal lipids play an important role in excess fetal growth. During pregnancy, maternal lipid profiles, including total cholesterol (TC), triglyceride (TG), low-density lipoprotein cholesterol (LDL-c), and high-density lipoprotein cholesterol (HDL-c), are taken up by placenta and primarily provide energy for maternal metabolism and fetal development (3, 4). To adapt to maternal-fetal physiology, maternal lipid levels rise progressively throughout gestation, suggesting the importance of these metabolic changes in fetal development (5).

Overweight and obesity in women of reproductive age, which are related to increased maternal lipid levels in pregnancy (6), keep increasing in China (7). However, hyperlipidemia not only occurs in overweight or obese women but also in normal weight women during pregnancy. Maternal hyperlipidemia has a variety of effects on intrauterine fetal growth and could significantly impact perinatal outcomes (8–12). Elevated TG concentrations in plasma may contribute to increased risks of impaired glucose tolerance and gestational diabetes (13, 14). Moreover, several studies have shown that higher TG and oxidized LDL were associated with preeclampsia (15, 16). Besides pregnancy complications, maternal hyperlipidemia may predict adverse birth outcomes as well, including preterm birth, large for gestational age (LGA), and macrosomia (11, 17). Beyond pregnancy, women with gestational hyperlipidemia were prone to metabolic syndrome and cardiovascular disease (18, 19). Thus, it is important to control maternal lipid concentrations in an optimal range for women in all body mass index (BMI) groups. However, there is no consensus on optimal normal ranges for lipids in pregnant women.

Birth weight is an important outcome reflecting intrauterine conditions and predicting short- and long-term morbidities. LGA and macrosomia indicate excess intrauterine weight gain and are related to adverse obstetrical outcomes such as postpartum hemorrhages, traumatic deliveries, and still birth (20, 21). LGA and macrosomia infants were also prone to diabetes and obesity in adulthood and childhood (22, 23). On the other hand, small for gestational age (SGA) and low birth weight (LBW) infants had higher incidence of hypoxic ischemic encephalopathy, seizures, neonatal sepsis (21) and associated with stroke, kidney disease, hypertension, and depression in later life (24, 25). Therefore, understanding the effect of maternal lipid profile on fetal growth is necessary for optimizing birth outcomes and subsequently decreasing the prevalence of numerous diseases beyond infancy.

So far, most studies focused on the impact of maternal lipid profile in the second and third trimesters during pregnancy on birth outcomes (26, 27). However, it would be ideal if high-risk women could be identified as early as possible. So, our study aims to shed more light on the association between maternal lipid profiles in the first trimester and birth weight and adverse birth outcomes. In addition, we attempted to find a reference value of maternal lipid profile considering the prevalence of LGA and macrosomia, which could be potentially applied to screen high-risk pregnant women for prenatal health care.

## Materials and methods

### Study population

This study recruited data from Obstetrics and Gynecology Hospital of Fudan University (Ob & Gyn Hospital) and International Peace Maternity and Child Health Hospital (IPMCHH), which are two major maternal health hospitals in Shanghai. Pregnant women who underwent prenatal health care since the first trimester and gave birth at the hospital from January 2018 to October 2020 were included in the analysis. Women who had a twin pregnancy or still birth, or with key medical data missing, namely, pre-pregnancy BMI and lipid profiles in early pregnancy, were excluded. All data, including serum TC, TG, LDL-c, HDL-c concentrations in early pregnancy, and birth information were collected. This study has been approved by Ob & Gyn Hospital (No. 2021-90) and IPMCHH Ethical committees (No. GKLW2019-05).

### Data collection and measures

All data were collected by in-person interviews during hospital visits and medical records. Maternal sociographic characteristics included residence, occupation, maternal age at birth, marital status, education, insurance status, and consumption of alcohol and cigarettes. Maternal pre-pregnancy weight was self-reported by enrolled women, and pre-pregnancy BMI (kg/m^2^) was calculated as pre-pregnancy weight (kilograms) divided by the square of height (meters). According to WHO classification, pre-pregnancy BMI was categorized into underweight (< 18.5), normal weight (18.5–24.9), overweight (25.0–29.9), and obesity (≥30) (28). Information related to pregnancy complications, including gestational diabetes, gestational hypertension disorders, intrahepatic cholestasis of pregnancy, and mode of conception were ascertained from medical records.

Fasting venous blood samples were drawn during 7:00 a.m. and 9:00 a.m. at the first prenatal visit during 8 to 13 gestational weeks. The lipid profiles were tested by the biochemical laboratory of IPMCHH and Ob & Gyn Hospital. Serum TC and TG concentrations were determined by GPO-POD method with a commercial enzymatic colorimetric assay (Beckman Coulter, CA, USA and Fujifilm, Osaka, Japan) and Beckman AU5800 analyzer and HITACHI 7600. LDL-c and HDL-c were examined by the direct method with a commercial reagent (Beckman Coulter, CA, USA and SEKISUI, TX, USA) and Beckman AU5800 analyzer and HITACHI 7600.

### Birth outcomes

In this study, birth weight and risks of LGA and macrosomia of singleton live births were the primary outcomes. Birth weight was standardized based on gestational age at birth (29). Data on birth weight, fetal sex, and gestational age were collected in the medical records at delivery. LGA was defined as an infant with birth weight larger than the 10^th^ percentile for his/her gestational age and sex, whereas small for gestational age (SGA) as smaller than the 10^th^ percentile (30). Macrosomia was diagnosed when the newborn weighed more than 4,000 g, whereas LBW was diagnosed with a birth weight of less than 2,500 g (31).

### Statistical analysis

Distributions of maternal TG, TC, LDL-c, and HDL-c concentrations were right-skewed. Therefore, the concentrations of lipids were natural log-transformed to improve the normality of their distributions (32). We conducted multiple linear regression models to evaluate the association between maternal lipids and neonatal birth weight. Ln transformed lipid concentrations were divided into quartiles, and >75^th^ percentile was defined as reference points of high lipid groups. Multiple logistic regression models were used to estimate odds ratios (ORs) and 95% confidential intervals (CIs) for the association between TC, TG, HDL-c, and LDL-c with LGA and SGA. Subgroup analyses were performed according to maternal pre-pregnancy BMI ranges. Furthermore, we investigated the combined effects of pre-pregnancy BMI and lipids in early pregnancy on LGA and macrosomia by adding a product interaction term of pre-pregnancy BMI × lipid concentrations (TC, TG, HDL-c, and LDL-c) in the models. Heat maps were constructed to exhibit the differences based on combinations of pre-pregnancy BMI and maternal TC, TG, HDL-c, LDL-c concentrations (red represents high incidence and blue represents low incidence). Confounders were included if they were previously reported in researches or were found correlated with the primary outcome. In our analyses, all the birth outcome models were adjusted for potential confounders, including maternal pre-pregnancy BMI (except in subgroup analyses), age at birth, mode of conception, parity, education attainment, consumption of cigarettes, infant sex, gestational diabetes, and gestational hypertension disorders. For confounders with missing data, multiple imputations were used based on the Markov chain Monte Carlo method. All analyses were performed using R software (version 4.0.4) with the “rms,” “mice,” and “visreg,” packages.

## Results

### Study population

A total of 57,516 women with live singleton deliveries were included in this study, and their descriptive characteristics were shown in **Table 1**. The mean age of mothers was 31.16 years, and the mean BMI was 21.29. Gestational diabetes and hypertension disorders affected 13.7 and 4.8% enrolled women. Among the infants, 51.7% were male and 5.1% were preterm birth. The mean value (SD) of birth weight was 3321.52 (453.25) g, with LGA, SGA, macrosomia, and LBW proportions of 18.1, 2.3, 5.7, and 3.3%, respectively.

**Table 1:** Descriptive statistics of study population.

Maternal characteristics	(*n* = 57516)
Age, Mean ± SD, years	31.16 ± 3.95
Pre-gestational BMI, Median (95% CI), kg/m^2^	20.8 (16.9, 28.3)
Gestational diabetes (%)	7875 (13.7)
Gestational hypertension disorders	
Gestational hypertension (%)	1284 (2.2)
Pre-eclampsia (%)	1070 (1.9)
Eclampsia (%)	404 (0.7)
Intrahepatic cholestasis of pregnancy	447 (0.8)
Parity	
Nullipara (%)	38182 (66.4)
Primi or multipara (%)	19334 (33.6)
**Fetal characteristics**	
Fetal sex	
Male (%)	29709 (51.7)
Female (%)	27807 (48.3)
Birth weight, Mean ± SD, Kg	3,321.52 ± 453.25
LBW (%)	1907 (3.3)
Macrosomia (%)	3258 (5.7)
SGA (%)	1320 (2.3)
LGA (%)	10390 (18.1)
Preterm birth (%)	
Very preterm	307 (0.5)
Late preterm	2664 (4.6)

SD, standard deviation; LBW, low birth weight; SGA, small for gestational age; LGA, large for gestational age.

The mean levels of TC, TG, LDL-c, and HDL-c in early pregnancy were 4.56 (1.37–12.79) mmol/L, 1.42 (0.10–13.11) mmol/L, 2.29 (0.51–9.58) mmol/L, and 1.58 (0.38–3.45) mmol/L, respectively. Their 75^th^ percentiles were 5.02 mmol/L, 1.67 mmol/L, 2.99 mmol/L, and 2.01 mmol/L (**Table 2**).

**Table 2:** Quartiles of maternal lipid profiles in first trimester (mmol/L).

	Mean ± SD	25th	50th	75th
TC	4.56 ± 0.83	3.99	4.48	5.02
TG	1.42 ± 0.65	0.98	1.29	1.67
LDL-c	2.29 ± 0.63	2.19	2.59	2.99
HDL-c	1.58 ± 0.52	1.21	1.67	2.01

TC, total cholesterol; TG, triglyceride; LDL-c, low-density lipoprotein cholesterol; HDL-c, high-density lipoprotein cholesterol.

### Maternal lipid profile and birth weight

**Figure 1:**
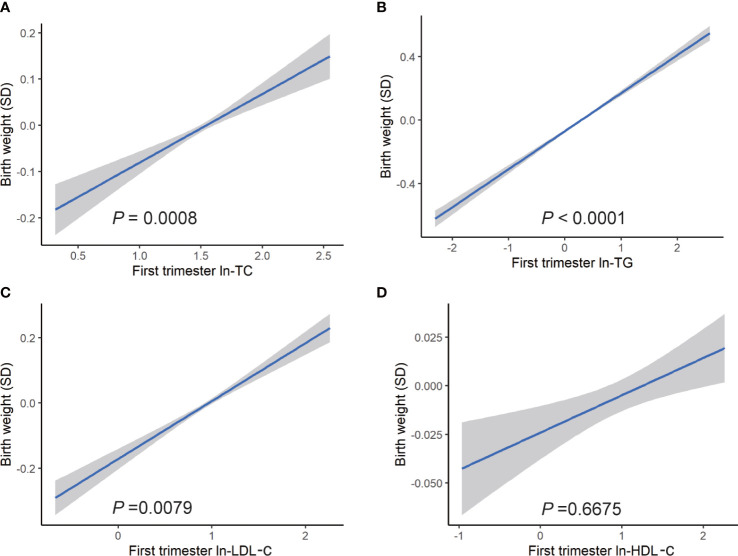
Association between ln transformed maternal lipid profiles in early pregnancy and birth weight. Linear regression models for **(A)** ln-TC, **(B)** ln-TG, **(C)** ln-LDL-c, **(D)** ln-HDL-c, and birth weight plotted as predicted mean with 95% CIs. Analyses were adjusted for maternal pre-pregnancy body mass index, age, mode of conception, parity, education, consumption of cigarettes, infant sex, gestational diabetes, and gestational hypertension disorders.

The association between maternal lipid concentrations in early pregnancy and birth weight was presented in **Figure 1**. After adjusting for confounders, the results displayed a significant positive relationship between maternal TC, TG, and LDL-c concentrations and birth weight. Each unit of natural log (ln) increase in TC, TG, and LDL-c was associated with 0.086 (95% CI: 0.036, 0.136) SD, 0.159 (95% CI: 0.136, 0.181) SD, and 0.071 (95% CI: 0.031, 0.111) increase in birth weight. However, we observed no association between ln-transformed HDL-c and birth weight.

Subgroup analyses showed a similar association. Ln-TG showed a significant association with birth weight in all BMI subgroups, whereas there were no visible association between birth weight with ln-TC nor ln-LDL-c in pre-pregnancy overweight women. In addition, in normal weight and obesity groups, we did not find any correlation between ln-LDL-c and birth weight (**Table 3**). In fetal sex subgroup analysis, a similar relationship was observed, whereas ln-TC showed a significant positive association with birth weight in male fetus but not female fetus.

**Table 3:** The association between blood metabolic markers in early pregnancy and fetal birth weight by different BMI groups.

	β (95% CI)^a^	*P*
**Normal**		
TC	0.06 (0.00, 0.12)	0.0357*
TG	0.15 (0.12, 0.17)	<0.0001*
LDL-c	0.04 (-0.02, 0.10)	0.1980
HDL-c	-0.01 (-0.03, 0.01)	0.4571
**Underweight**		
TC	0.23 (0.11, 0.36)	0.0003*
TG	0.16 (0.10, 0.22)	<0.0001*
LDL-c	0.21 (0.12, 0.29)	<0.0001*
HDL-c	-0.01 (-0.05, 0.04)	0.8774
**Overweight**		
TC	-0.01 (-0.19, 0.16)	0.8785
TG	0.20 (0.13, 0.28)	<0.0001*
LDL-c	-0.07 (-0.19, 0.05)	0.2839
HDL-c	0.02 (-0.17, 0.21)	0.82401
**Obesity**		
TC	0.52 (-0.04, 1.08)	0.0698
TG	0.41 (0.18, 0.65)	0.0007*
LDL-c	0.30 (-0.10, 0.70)	0.1358
HDL-c	0.02 (-0.17, 0.21)	0.82401

a Adjusted for maternal age, mode of conception, parity, education attainment, consumption of cigarettes, infant sex, gestational diabetes, and gestational hypertension disorders.

LGA, large for gestational age; TC, total cholesterol; TG, triglyceride; LDL-c, low density lipoprotein cholesterol; HDL-c, high-density lipoprotein cholesterol; CI, confidential interval. * A significant association was found statistically.

### Maternal lipid profile and birth outcomes

**Figure 2:**
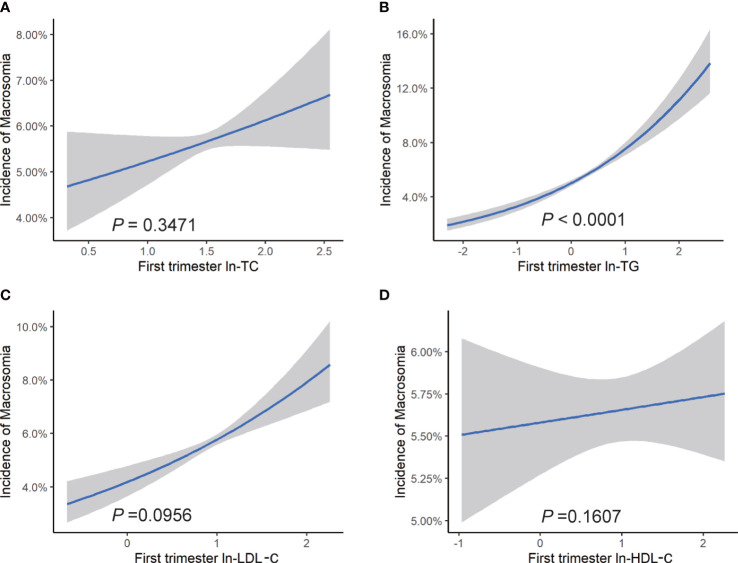
Risk of macrosomia associated with maternal lipid profiles in early pregnancy. Logistic regression models for ln transformed **(A)** TC, **(B)** TG, **(C)** LDL-c, **(D)** HDL-c, and macrosomia, expressed as predicted mean with 95% CIs. Analyses were adjusted for maternal pre-pregnancy BMI, age, mode of conception, parity, education, consumption of cigarettes, infant sex, gestational diabetes, and gestational hypertension disorders.

**Figure 3:**
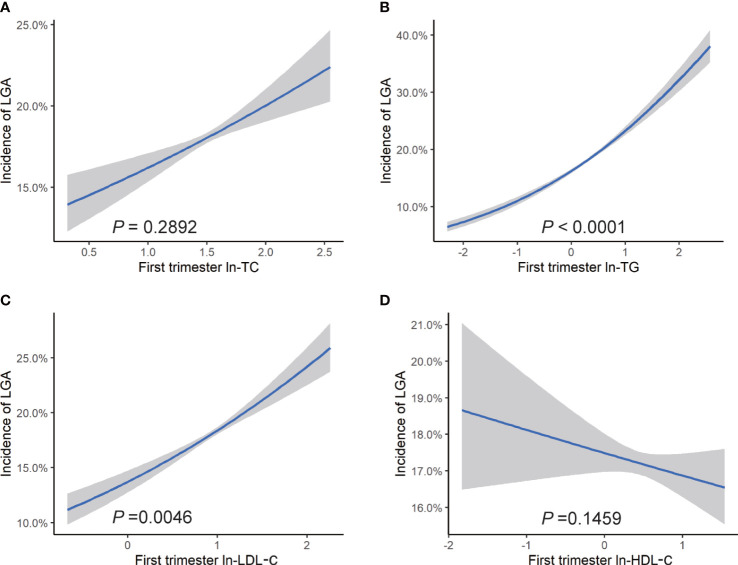
Risk of LGA associated with maternal lipid profiles in early pregnancy. Logistic regression models for ln transformed **(A)** TC, **(B)** TG, **(C)** LDL-c, **(D)** HDL-c, and LGA, expressed as predicted mean with 95% CIs. Analyses were adjusted for maternal pre-pregnancy BMI, age, mode of conception, parity, education, consumption of cigarettes, infant sex, gestational diabetes, and gestational hypertension disorders.

After adjustment for confounders, we observed a positive association between each unit increase of maternal ln-TG in the first trimester and odds of macrosomia (OR = 1.37, 95% CI: 1.24, 1.52) (**Figure 2**). The increase of ln-TG (OR = 1.33, 95% CI: 1.25, 1.42) and ln-LDL-c (OR = 1.12, 95% CI:1.02, 1.24) in early pregnancy also exhibited a positive association with the risks of LGA (**Figure 3**). Moreover,while first trimester lipid profiles was not associated with LBW (**Figure s1**), ln-TC (OR = 0.67, 95% CI: 0.49, 1.00), ln-TG (OR = 0.73, 95% CI: 0.62, 0.85) and HDL-c (OR = 0.83, 95% CI: 0.68, 1.00) were inversely associated with the prevalence of SGA (**Figure s2**).

In subgroup analyses based on pre-pregnancy BMI ranges, the relationship between ln-TG and LGA was observed in all BMI categories, with ORs ranging from 1.24 to 1.72. However, no significant association between first trimester ln-TG and the incidence of macrosomia was observed in pre-pregnancy overweight and obese women. Similarly, we did not find any correlation between ln-LDL-c and LGA in overweight and obese women, whereas a negative association was observed between ln-HDL-c and odds of LGA in underweight women. In addition, the prevalence of SGA was shown to be inversely related to ln-TG in all subgroups and negatively associated with HDL-c in pre-pregnancy underweight group. However, we did not find any association between lipid levels in early pregnancy with the risk of LBW in none of the groups (**Table s1**).

Compared with women of normal serum lipid concentrations, women with high-maternal TC (> 5.02 mmol/L) and TG (> 1.67 mmol/L) exhibited significant increase risks of LGA (OR = 1.07, 95% CI: 1.00, 1.14 and OR = 1.21, 95% CI: 1.15, 1.28), and women in high TG and LDL-c (> 2.99 mmol/L) group showed elevated risks of macrosomia (OR = 1.20, 95% CI: 1.10, 1.31 and OR = 1.09, 95% CI: 1.10, 1.19). We also observed decreased prevalence of SGA in high TG group (OR = 0.71, 95% CI: 0.61, 0.83) (**Table 4**).

**Table 4:** Maternal lipid profiles and risks of LGA, macrosomia, LBW, and SGA by BMI groups.

	All		Normal weight		Underweight		Overweight		Obesity	
	**≤75th**	**> 75th**	**≤75th**	**> 75th**	**≤75th**	**> 75th**	**≤75th**	**> 75th**	**≤75th**	**> 75th**
**TC**										
**LGA**										
*n* (%)	7131 (16.5)	2543 (17.7)	5400 (16.5)	1916 (17.6)	544 (8.7)	168 (9.5)	1007 (27.4)	381 (25.8)	180 (35.8)	78 (36.3)
OR (95% CI)	1.00 (reference)	1.07 (1.00, 1.14)	1.00 (reference)	1.09 (1.03, 1.17)	1.00 (reference)	1.08 (0.87, 1.33)	1.00 (reference)	0.91 (0.78, 1.05)	1.00 (reference)	0.94 (0.66, 1.36)
**Macrosomia**										
*n* (%)	2407 (5.6)	851 (5.9)	1792 (5.5)	621 (5.7)	132 (2.1)	53 (3)	408 (11.1)	148 (10.0)	75 (14.9)	29 (13.5)
OR (95% CI)	1.00 (reference)	1.05 (0.96, 1.16)	1.00 (reference)	1.09 (0.98, 1.22)	1.00 (reference)	1.39 (0.93, 2.07)	1.00 (reference)	0.93 (0.74, 1.16)	1.00 (reference)	0.88 (0.53, 1.48)
**LBW**										
*n* (%)	1407 (3.3)	500 (3.5)	1000 (3.1)	348 (3.2)	243 (3.9)	72 (4.1)	146 (4)	64 (4.3)	18 (3.6)	16 (7.4)
OR (95% CI)	1.00 (reference)	0.98 (0.83, 1.16)	1.00 (reference)	0.93 (0.76, 1.14)	1.00 (reference)	1.00 (0.67, 1.48)	1.00 (reference)	1.07 (0.62, 1.84)	1.00 (reference)	3.06 (0.77, 12.08)
**SGA**										
*n* (%)	1002 (2.3)	318 (2.2)	708 (2.2)	229 (2.1)	235 (3.8)	60 (3.4)	51 (1.4)	26 (1.8)	8 (1.6)	3 (1.4)
OR (95% CI)	1.00 (reference)	0.89 (0.76, 1.03)	1.00 (reference)	0.87 (0.73, 1.04)	1.00 (reference)	0.87 (0.62, 1.22)	1.00 (reference)	1.23 (0.72, 2.09)	1.00 (reference)	0.33 (0.04, 3.05)
**TG**										
**LGA**										
*n* (%)	6693 (15.5)	2981 (20.8)	5204 (15.8)	2112 (19.7)	571 (8.5)	141 (10.9)	793 (25.6)	595 (29.0)	125 (30.7)	133 (42.8)
OR (95% CI)	1.00 (reference)	1.21 (1.15, 1.28)	1.00 (reference)	1.29 (1.22, 1.38)	1.00 (reference)	1.23 (0.98, 1.55)	1.00 (reference)	1.15 (1.00, 1.31)	1.00 (reference)	1.61 (1.15, 2.32)
**Macrosomia**										
*n* (%)	2267 (5.3)	991 (6.9)	1739 (5.3)	674 (6.3)	142 (2.1)	43 (3.3)	330 (10.7)	226 (11)	56 (13.8)	48 (15.4)
OR (95% CI)	1.00 (reference)	1.20 (1.10, 1.31)	1.00 (reference)	1.29 (1.16, 1.43)	1.00 (reference)	1.51 (0.98, 2.31)	1.00 (reference)	1.13 (0.93, 1.38)	1.00 (reference)	1.24 (0.79, 1.96)
**LBW**										
*n* (%)	1388 (3.2)	519 (3.6)	989 (3)	359 (3.4)	265 (3.9)	50 (3.9)	117 (3.8)	93 (4.5)	17 (4.2)	17 (5.5)
OR (95% CI)	1.00 (reference)	1.05 (0.99, 1.10)	1.00 (reference)	1.05 (0.99, 1.11)	1.00 (reference)	0.79 (0.49, 1.26)	1.00 (reference)	1.04 (0.91, 1.19)	1.00 (reference)	1.06 (0.73, 1.53)
**SGA**										
*n* (%)	1036 (2.4)	284 (2)	730 (2.2)	207 (1.9)	247 (3.7)	48 (3.7)	51 (1.6)	26 (1.3)	8 (2.0)	3 (1.0)
OR (95% CI)	1.00 (reference)	0.71 (0.61, 0.83)	1.00 (reference)	0.87 (0.73, 1.05)	1.00 (reference)	1.18 (0.83, 1.67)	1.00 (reference)	0.66 (0.38, 1.12)	1.00 (reference)	0.2 (0.02, 1.97)
**LDL**										
**LGA**										
*n* (%)	6988 (16.3)	2679 (18.4)	5332 (16.4)	1978 (17.9)	572 (8.7)	140 (9.5)	934 (27.9)	453 (25.1)	150 (35.9)	108 (36)
OR (95% CI)	1.00 (reference)	1.04 (0.99, 1.10)	1.00 (reference)	1.11 (1.05, 1.18)	1.00 (reference)	1.15 (0.94, 1.41)	1.00 (reference)	0.89 (0.71, 1.02)	1.00 (reference)	1.06 (0.76, 1.47)
**Macrosomia**										
n (%)	2329 (5.4)	926 (6.3)	1756 (5.4)	655 (5.9)	142 (2.2)	43 (2.9)	372 (11.1)	183 (10.1)	59 (14.1)	45 (15)
OR (95% CI)	1.00 (reference)	1.09 (1.00, 1.19)	1.00 (reference)	1.15 (1.04, 1.27)	1.00 (reference)	1.56 (1.08, 2.25)	1.00 (reference)	0.94 (0.77, 1.15)	1.00 (reference)	1.25 (0.80, 1.97)
**LBW**										
*n* (%)	1369 (3.2)	529 (3.6)	960 (2.9)	381 (3.5)	263 (4)	51 (3.5)	128 (3.8)	81 (4.5)	18 (4.3)	16 (5.3)
OR (95% CI)	1.00 (reference)	1.01 (0.97, 1.07)	1.00 (reference)	0.99 (0.82, 1.2)	1.00 (reference)	0.76 (0.5, 1.15)	1.00 (reference)	0.99 (0.6, 1.62)	1.00 (reference)	1.38 (0.37, 5.18)
**SGA**										
*n* (%)	986 (2.3)	328 (2.2)	698 (2.1)	234 (2.1)	241 (3.7)	53 (3.6)	41 (1.2)	36 (2)	6 (1.4)	5 (1.7)
OR (95% CI)	1.00 (reference)	0.86 (0.68, 1.1)	1.00 (reference)	1.00 (0.85, 1.18)	1.00 (reference)	0.85 (0.61, 1.18)	1.00 (reference)	1.59 (0.97, 2.6)	1.00 (reference)	1.71 (0.37, 7.88)
	**≤ 25th**	**> 25th**	**≤ 25th**	**> 25th**	**≤ 25th**	**> 25th**	**≤ 25th**	**> 25th**	**≤ 25th**	**> 25th**
**HDL**										
**LGA**										
*n* (%)	2268 (15.9)	7406 (17.1)	1686 (15.9)	5630 (17.1)	167 (8.3)	545 (9.1)	337 (24.2)	1051 (28)	78 (33.1)	180 (37.3)
OR (95% CI)	1.00 (0.94, 1.07)	1.00 (reference)	1.03 (0.96, 1.11)	1.00 (reference)	1.10 (0.88, 1.38)	1.00 (reference)	0.91 (0.78, 1.08)	1.00 (reference)	1.10 (0.75, 1.61)	1.00 (reference)
**Macrosomia**										
*n* (%)	839 (5.9)	2419 (5.6)	625 (5.9)	1788 (5.4)	52 (2.6)	133 (2.2)	137 (9.8)	419 (11.1)	25 (10.6)	79 (16.4)
OR (95% CI)	1.01 (0.91, 1.11)	1.00 (reference)	1.09 (0.97, 1.22)	1.00 (reference)	1.09 (0.71, 1.67)	1.00 (reference)	0.89 (0.70, 1.13)	1.00 (reference)	0.59 (0.34, 1.02)	1.00 (reference)
**LBW**										
*n* (%)	443 (3.1)	1464 (3.4)	310 (2.9)	1038 (3.1)	64 (3.2)	251 (4.2)	58 (4.2)	152 (4)	11 (4.7)	23 (4.8)
OR (95% CI)	0.90 (0.71, 1.13)	1.00 (reference)	1.05 (0.84, 1.31)	1.00 (reference)	0.94 (0.61, 1.46)	1.00 (reference)	0.65 (0.35, 1.21)	1.00 (reference)	0.24 (0.04, 1.54)	1.00 (reference)
**SGA**										
*n* (%)	378 (2.7)	942 (2.2)	268 (2.5)	669 (2)	88 (4.4)	207 (3.4)	18 (1.3)	59 (1.6)	4 (1.7)	7 (1.5)
OR (95% CI)	0.83 (0.68, 1.02)	1.00 (reference)	1.11 (0.93, 1.33)	1.00 (reference)	1.28 (0.92, 1.77)	1.00 (reference)	0.54 (0.29, 1)	1.00 (reference)	1.46 (0.27, 7.98)	1.00 (reference)

Data are shown as OR and 95% CI, adjusted for maternal age, mode of conception, parity, education attainment, consumption of cigarettes, infant sex, gestational diabetes, and gestational hypertension disorders. LGA, large for gestational age; LBW, low birth weight; SGA, small for gestational age; TC, total cholesterol; TG, triglyceride; LDL-c, low-density lipoprotein cholesterol; HDL-c, high-density lipoprotein cholesterol.

### Combined effects of lipid concentrations and pre-pregnancy BMI on the risks of LGA and macrosomia

**Figure 4:**
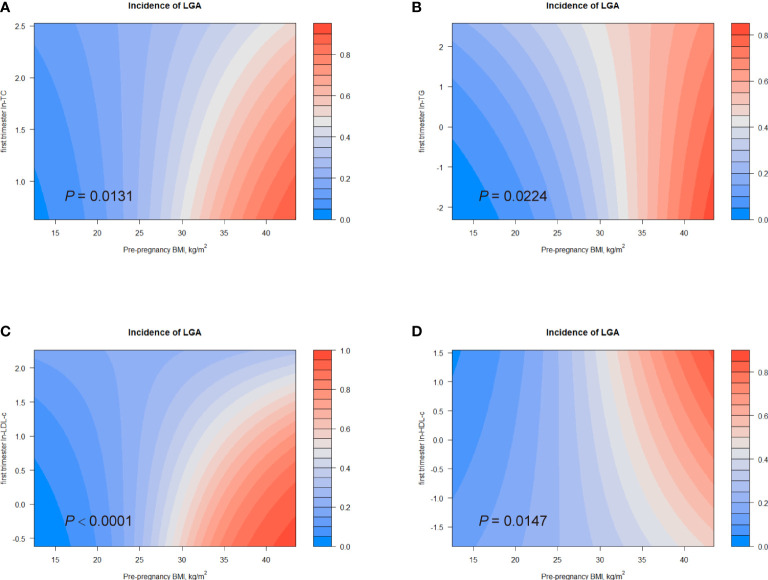
Combined effects of maternal pre-pregnancy BMI and lipid profiles in early pregnancy on incidence of LGA. Heat map for the correlation of incidence of LGA (red represents increased risks of LGA, blue represents decreased risks of LGA) according to the interaction of pre-pregnancy BMI and **(A)** ln-TC, **(B)** ln-TG, **(C)** ln-LDL-c, or **(D)** ln-HDL-c. Analyses were adjusted for maternal pre-pregnancy BMI, age, mode of conception, parity, education, consumption of cigarettes, infant sex, gestational diabetes and gestational hypertension disorders.

**Figure 5:**
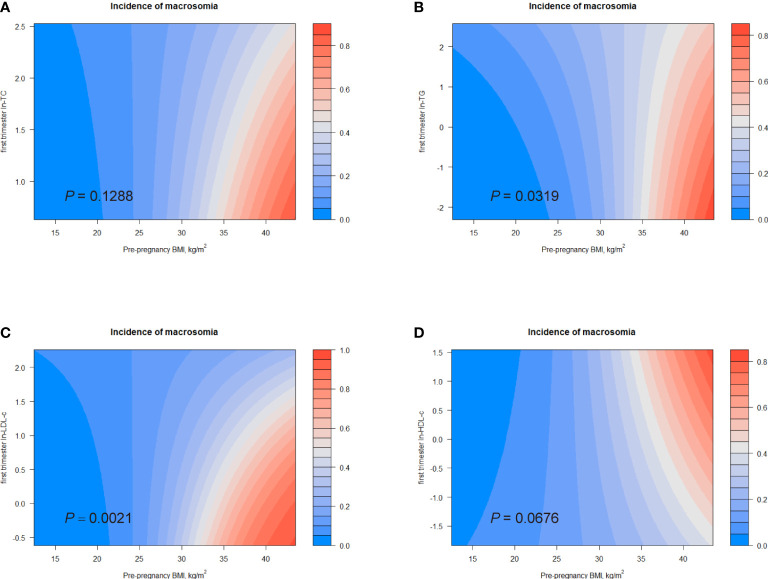
Combined effects of maternal pre-pregnancy BMI and lipid profiles in early pregnancy on incidence of macrosomia. Heat map for the correlation of incidence of macrosomia (red represents increased risks of LGA, blue represents decreased risks of LGA) according to the interaction of pre-pregnancy BMI and **(A)** ln-TC, **(B)** ln-TG, **(C)** ln-LDL-c, or **(D)** ln-HDL-c. Analyses were adjusted for maternal pre-pregnancy BMI, age, mode of conception, parity, education, consumption of cigarettes, infant sex, gestational diabetes, and gestational hypertension disorders.

Increased BMI was believed to be companied by unfavorable lipid levels characterized by high concentrations of TG, TC and LDL-c, and low concentrations of HDL-c (**Table s2**) **(** 6). Additionally, we observed a positive association between pre-pregnancy BMI and birth weight (**Figure s3**). Therefore, we investigated the combined effects of first trimester lipid profiles and pre-pregnancy BMI on birth outcomes. **Figures 4**, **5** displayed heat maps for the combined association of pre-pregnancy BMI (*x*-axis) (ranged from 15.2 to 34.8 kg/m^2^) and maternal ln-TC, ln-TG, ln-HDL-c, or ln-LDL-c (*y*-axis) in the first trimester with the incidence of LGA (%) and macrosomia (%) (*z*-axis; red represents higher incidence and blue represents lower incidence).

Considerable differences in the incidence of LGA according to the combination of pre-pregnancy BMI and first trimester ln-TG were identified (*P* for interaction = 0.0224; **Figure 4**). The interactive effect also showed that, in women with pre-pregnancy BMI less than 35 kg/m^2^, increased ln-TG in early pregnancy and pre-pregnancy BMI was associated with higher risks of LGA. A similar effect was observed in the combination of pre-pregnancy BMI and ln-TC levels (*P* for interaction = 0.0131) (**Figure 4**). The interaction between pre-pregnancy BMI and ln-LDL-c was also significantly identified (*P* for interaction < 0.0001). While ln-LDL-c displayed a positive association with LGA in women with pre-pregnancy BMI less than 25 kg/m^2^, a negative relationship was observed in obese women (BMI > 30) (**Figure 4**). Differently, increasing HDL-c indicated lower risks of LGA in women with pre-pregnancy BMI < 35 kg/m^2^ (**Figure 4**). Similarly, significant combined effects of ln-TG/ln-LDL-c and pre-pregnancy BMI on macrosomia were observed (ln-TG, *P* for interaction = 0.0319; ln-LDL-c, *P* for interaction = 0.0021, **Figures 5B**). Like the effect on LGA, ln-LDL-c exhibited a negative association with macrosomia in obese women, too. However, we did not observe any significant combined effect on SGA or LBW (**Figures s3, s4**).

## Discussion

In this large retrospective study, we found that elevated maternal lipids in early pregnancy, especially TG, were significantly associated with higher birth weight and increased risks of LGA and macrosomia. We also proposed 75^th^ percentile as reference points for first trimester lipid concentrations based on a large Chinese population. Our results firstly suggested a considerable combined effect of pre-pregnancy BMI and first trimester TG on birth outcomes, which may provide valuable information on early pregnancy screening and contribute to perinatal health care.

To meet the increasing physiological demands of fetal development, maternal serum lipid concentrations during gestation are generally higher than that in non-pregnant status. Recently, several studies displayed a discernible decrease of lipid levels in the first 6 weeks of pregnancy (33, 34). Later on, the lipid levels keep raising and peak at late third trimester (35). Clinicians usually use the lipid criteria for non-pregnant people to evaluate gestational lipid levels considering that an accurate normal lipid concentration range especially for pregnant women is lacking. However, pregnancy is a vulnerable period during which both mothers and infants are susceptible to adverse lipid environment; thus, a reference range for lipids in pregnant women is urgently needed. As maternal overnutrition is an increasing issue in Shanghai instead of maternal undernutrition, our study aimed to find out lipid reference values to prevent excessive birth weight. Based on a large sample size in two centers in China, we compared the risks of LGA and macrosomia in different percentile groups and we recommended that the 75^th^ percentile of lipid concentrations in the current study could serve as a reference point predicting the prevalence of adverse birth outcomes. In pregnant women whose TG levels were below 75^th^, the risks of LGA and macrosomia decreased from 18.7 and 5.7% to 15.3 and 5.3%, compared with 16.7 and 5.5% in women whose TG was below 95^th^ percentile. In our study, the reference points of 75^th^ in early pregnancy were slightly lower than the lipid criteria for non-pregnant people (95^th^ percentile) considering the vulnerability of pregnant women. Because maternal serum lipid levels continue increasing in second and third trimesters, the elevated lipids in early pregnancy may forecast a more serious hyperlipidemia in late pregnancy and predict higher risks of large infants. Our reference values could be helpful for screening high-risk pregnant women in early pregnancy.

The influence of TG during pregnancy has gained some attention, and we found a stable association between TG with birth weight and adverse birth outcomes in all of the BMI groups in the current study. Our study is in line with several previous studies that reported a positive association between maternal TG concentrations in early pregnancy and higher birth weight and LGA (36–38). In a study proposed by *Wang et al.* in 2016, maternal TG concentrations in early pregnancy were divided into quartiles, and they did not observe any association between the highest quartile and an increased prevalence of LGA, whereas in the current study, the highest TG quartile showed a significantly higher risk of LGA (39). Because the 75^th^ percentile reported by *Wang et al.* was 1.40 mmol/L, which is lower than that in our study (1.67 mmol/L), the differences of lipid levels may contribute to the discrepancies in results. An obvious increase of first trimester TG concentrations was also noticed in Chinese pregnant women during the last 5 years, which may be related to the changes in diet structure (39). In addition, our study observed that increased TG levels in the first trimester were correlated with elevated risks of macrosomia, which was rarely studied previously. A negative association between TG levels and SGA was also found in the current study, which is consistent with several previous studies (12, 40). In a word, TG exhibited a significant relationship with birth weight and adverse birth outcomes, indicating that TG might play an important role in fetal growth. Additionally, our team previously found that first trimester TG could be a major predictor of gestational diabetes and was associated with preterm birth (17, 41), and others also suggested that high TG might induce gestational hypertension disorders and influence infant post-natal growth (37, 42), emphasizing the important role of TG during pregnancy. Our results supported that first trimester TG could predict adverse birth outcomes, thus the screening in early pregnancy is essential in prenatal health care.

Except for TG, first trimester TC levels exhibited a positive association with birth weight. However, we did not observe any significant association between TC and LGA nor macrosomia in both total population and subgroup analyses, which is consistent with previous researches (37, 43). While previous studies did not propose any association between maternal LDL-c levels and abnormal birth weight (44–46), we observed elevated LDL-c in the first trimester increased odds of LGA, but not macrosomia. In the current study, no detectable association between maternal HDL-c and birth weight or adverse birth outcomes was found as previously suggested in several studies (47, 48) (36, 49). The inconsistent results in different studies could be explained by different gestational weeks when the blood was collected and the variances in the study population.

Unfavorable lipid levels were considered to be related to increased pre-pregnancy BMI. Independent of lipid concentrations, maternal pre-pregnancy overweight, and obesity were reported to increase birth weight and the risks of relevant birth outcomes (50, 51), so we performed a subgroup analysis according to BMI categories. In all subgroups, the association between TG and birth weight and LGA was consistent, suggesting that TG might be a key factor influencing birth weight and predicting LGA. The higher concentrations of TG and smaller subgroup population may explain the inconsistent results in macrosomia. Our results also showed significantly increased risks of excessive birth weight in mothers with pre-pregnancy overweight and obesity (**Table 4**). Furthermore, the combined effects of pre-pregnancy BMI and lipid profiles on birth outcomes were studied, and significant differences were found according to the combination of pre-pregnancy BMI and first trimester TG levels. Our findings indicated that maternal pre-pregnancy BMI could influence gestational lipid levels and further affect birth weight. Taken together, we hypothesized that pre-pregnancy BMI along with first trimester TG would predict birth outcomes in large measure, and that pregnant women with elevated first trimester lipid concentrations and pre-pregnancy BMI should pay more attention to gestational weight management, including diet control, nutrition education, and moderate exercise. Surprisingly, we observed a negative association between ln-LDL-c with LGA and macrosomia in obese women in the combined effect analysis. As Vahratian et al. reported that the increasement of LDL-c levels during gestation in obese women was discernibly smaller than that in normal pregnancies, we assumed that the metabolic dysregulation and relatively low LDL-c concentrations in late pregnancy may account for the results (52).

Most previous studies focused on the effect of intrauterine lipid exposure in late pregnancy on birth weight, as it is conventionally believed that placental blood flow extensively increases to meet fetal growth and maternal circulating lipids directly impair placental vascular endothelium, leading to placental underperfusion and abnormal birth weight (53, 54). However, our study may indicate the important role of lipid levels in the first trimester. According to previous studies, elevated TG gives an increase in fatty acids, which may influence placental development and angiogenesis (42). In addition, fatty acids could act as growth factors and compete with hormones in binding to albumin, thus increase the free hormone levels such as sex hormones in circulation and subsequently impact intrauterine fetal growth (55). Moreover, maternal lipids in early pregnancy were found to be related to gestational complications such as gestational diabetes, which is a major contributor to LGA and macrosomia (56, 57).

The major strength of our study is the large study population from two centers and thorough and standardized medical records. In addition, the venous blood was drawn in a fasting state, which could reflect the lipid metabolic status better. Moreover, to our knowledge, this is one of the first studies to investigate the combined effects of pre-pregnancy BMI and maternal lipid profiles in early pregnancy on birth outcomes. However, the present study also has limitations, such as some self-reported data (pre-pregnancy weight); a rather small ratio for pre-pregnancy overweight and obese women; lack of some important data, such as maternal blood pressure levels and assessment of placenta function; and not so strict exclusion criteria, as we did not exclude the pregnant women with gestational complications including gestational diabetes and hypertensive disorders. Therefore, we performed a sensitivity analysis to show the relationship between maternal lipid levels and birth weight in women without these conditions and found similar results (**Table s4**.). Also, the clinical significance of the reference values proposed in this study remains unclear, and we appeal more studies focusing on this issue. In addition, as a retrospective study, the unbalanced baseline data may lead to potential bias, although we have adjusted for confounders in statistical analysis.

In conclusion, maternal first trimester lipid profiles, especially TG, were associated with higher birth weight and increased risks of LGA and macrosomia in different pre-pregnancy BMI categories. Additionally, lipids screening during early pregnancy and pre-pregnancy weight status assessment should be essential to filter mothers who are prone to having infants of elevated birth weight. More studies focusing on the effect of gestational lipid profiles are necessary considering maternal and fetal health.

## Data availability statement

The raw data supporting the conclusions of this article will be made available by the authors, without undue reservation.

## Ethics statement

Written informed consent was obtained from the individual(s) for the publication of any potentially identifiable images or data included in this article.

## Author contributions

Data interpretation, formal analysis, writing: S-MZ and H-QZ. Data curation: CL, CZ, and J-LY. Project administration, funding acquisition, supervision and revision of the manuscript: Y-TW and H-FH. All authors have read the manuscript and agreed to the submitted version of the manuscript.

## Funding

This research is supported by National Key Research and Development Program of China (2021YFC2700701), National Natural Science Foundation of China (81661128010, 82171686, 82001571, 82088102), the International Science and Technology Collaborative Fund of Shanghai (18410711800), Program of Shanghai Academic Research Leader (20XD1424100), Clinical Research Plan of Shanghai Shenkang Hospital Development Center (SHDC12018X17, SHDC12019107 and SHDC2020CR1008A) and Shanghai Frontiers Science Research Base of Reproduction and Development, Science and Technology Innovation Fund of Shanghai Jiao Tong University (YG2019GD04, YG2020YQ29) and CAMS Innovation Fund for Medical Sciences (2019-I2M-5-064). Natural Science Foundation of Shanghai (20ZR1463100), Collaborative Innovation Program of Shanghai Municipal Health Commission (2020CXJQ01), Outstanding Youth Medical Talents of Shanghai Rising Stars of Medical Talent Youth Development Program and Shanghai “Science and Technology Innovation Action Plan” Hong Kong, Macao, and Taiwan Science and Technology Cooperation Project (19410760100).

## Acknowledgments

We thank all the doctors and nurses who helped with recruitment for the study. We also thanked the pregnant women who participated in the current study.

## Conflict of interest

The authors declare that the research was conducted in the absence of any commercial or financial relationships that could be construed as a potential conflict of interest.

## Publisher’s note

All claims expressed in this article are solely those of the authors and do not necessarily represent those of their affiliated organizations, or those of the publisher, the editors and the reviewers. Any product that may be evaluated in this article, or claim that may be made by its manufacturer, is not guaranteed or endorsed by the publisher.
