# Supplementary material for: Maternal lipid profile during early pregnancy and birth weight: A retrospective study
Source: Front Endocrinol (Lausanne). 2022 Sep 15;13:951871. doi: 10.3389/fendo.2022.951871 (PMC9521310; doi:10.3389/fendo.2022.951871)
Supplement: Supplementary file 1 [file DataSheet_1.docx]

Supplementary Material: Maternal lipid profile during early pregnancy and birth weight: A retrospective study

Si-Meng Zhu^1,2,4^^†^, Han-Qiu Zhang^1,2,4†^, Cheng Li^3^, Chen Zhang^1,3^, Jia-Le Yu^1,2,4^, Yan-Ting Wu^,3*^, He-Feng Huang^1,2^^,3,4*^

# Supplementary Figures and Tables

Fig s1. Association between ln transformed maternal lipid profiles in early pregnancy and birth weight in male fetus.

Fig s2. Association between ln transformed maternal lipid profiles in early pregnancy and birth weight in female fetus.

Fig s3. Risk of LBW associated with maternal lipid profiles in early pregnancy.

Fig s4. Risk of SGA associated with maternal lipid profiles in early pregnancy.

Figure s5. Combined effects of maternal pre-pregnancy BMI and lipid profiles in early pregnancy on incidence of SGA.

Figure s6. Combined effects of maternal pre-pregnancy BMI and lipid profiles in early pregnancy on incidence of LBW.

Fig s7. The association between maternal pre-pregnancy BMI and birth weight.

Table s1. Odds ratio of Macrosomia, LGA, LBW and SGA with per-unit increase of blood lipid profiles (ln-transformed) by BMI groups.

Table s2. Maternal lipid profiles in different pre-pregnancy categories.

Table s3. Maternal lipid profiles and risks of LGA, macrosomia, SGA and LBW by BMI groups.

Table s4. Sensitivity analysis between matenal lipid profiles and birth weight (SD) after excluding GDM and gestational hypertention disorders population.


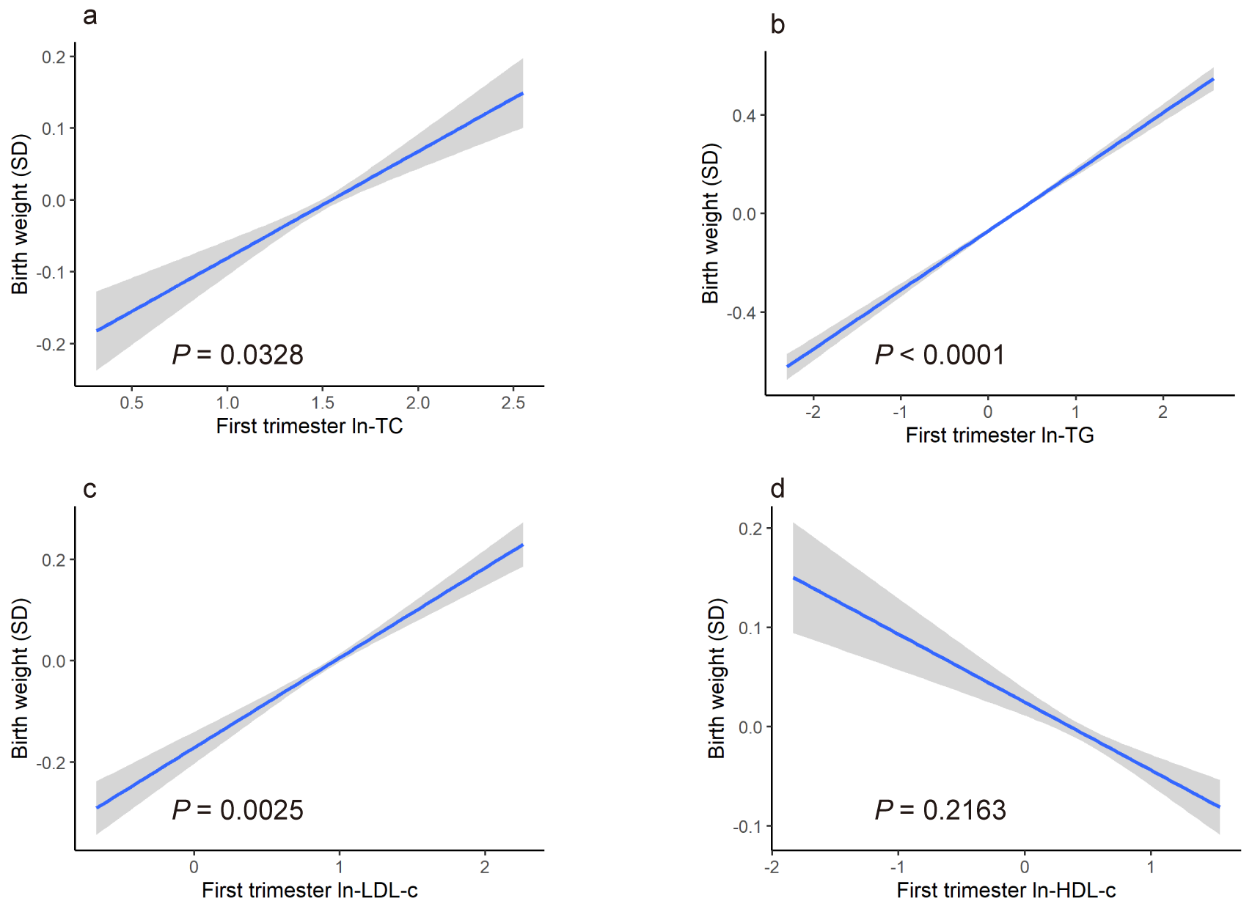


**Supplementary Figure 1.** **Association between ln transformed maternal lipid profiles in early pregnancy and birth weight in male fetus.** Linear regression models for (a) ln-TC, (b) ln-TG, (c) ln-LDL-c, (d) ln-HDL-c and birth weight plotted as predicted mean with 95% CIs. Analyses were adjusted for maternal pre-pregnancy body mass index, age, mode of conception, parity, education, consumption of cigarettes, gestational diabetes and gestational hypertension disorders.


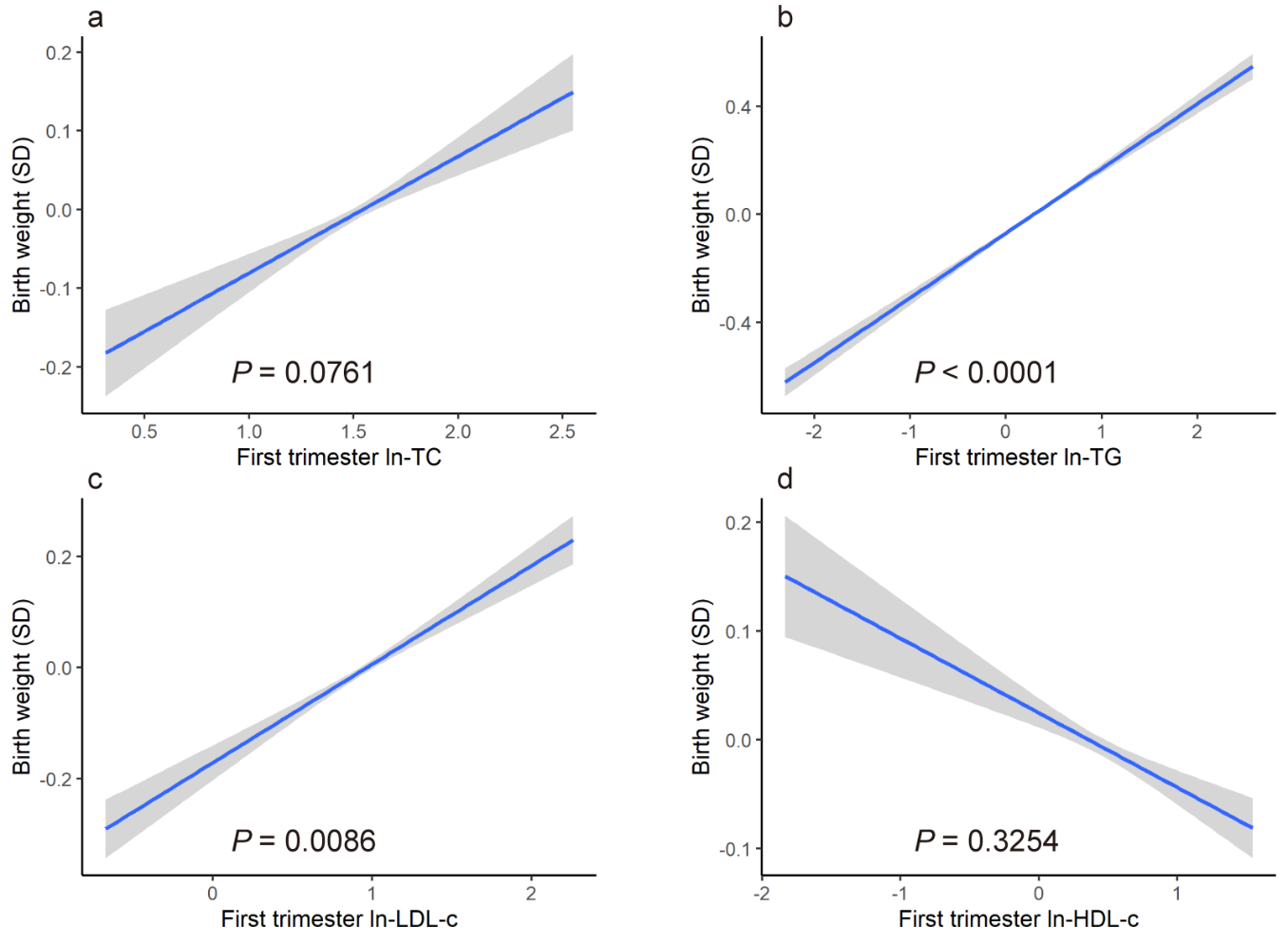


**Supplementary Figure 2.** **Association between ln transformed maternal lipid profiles in early pregnancy and birth weight in female fetus.** Linear regression models for (a) ln-TC, (b) ln-TG, (c) ln-LDL-c, (d) ln-HDL-c and birth weight plotted as predicted mean with 95% CIs. Analyses were adjusted for maternal pre-pregnancy body mass index, age, mode of conception, parity, education, consumption of cigarettes, gestational diabetes and gestational hypertension disorders.


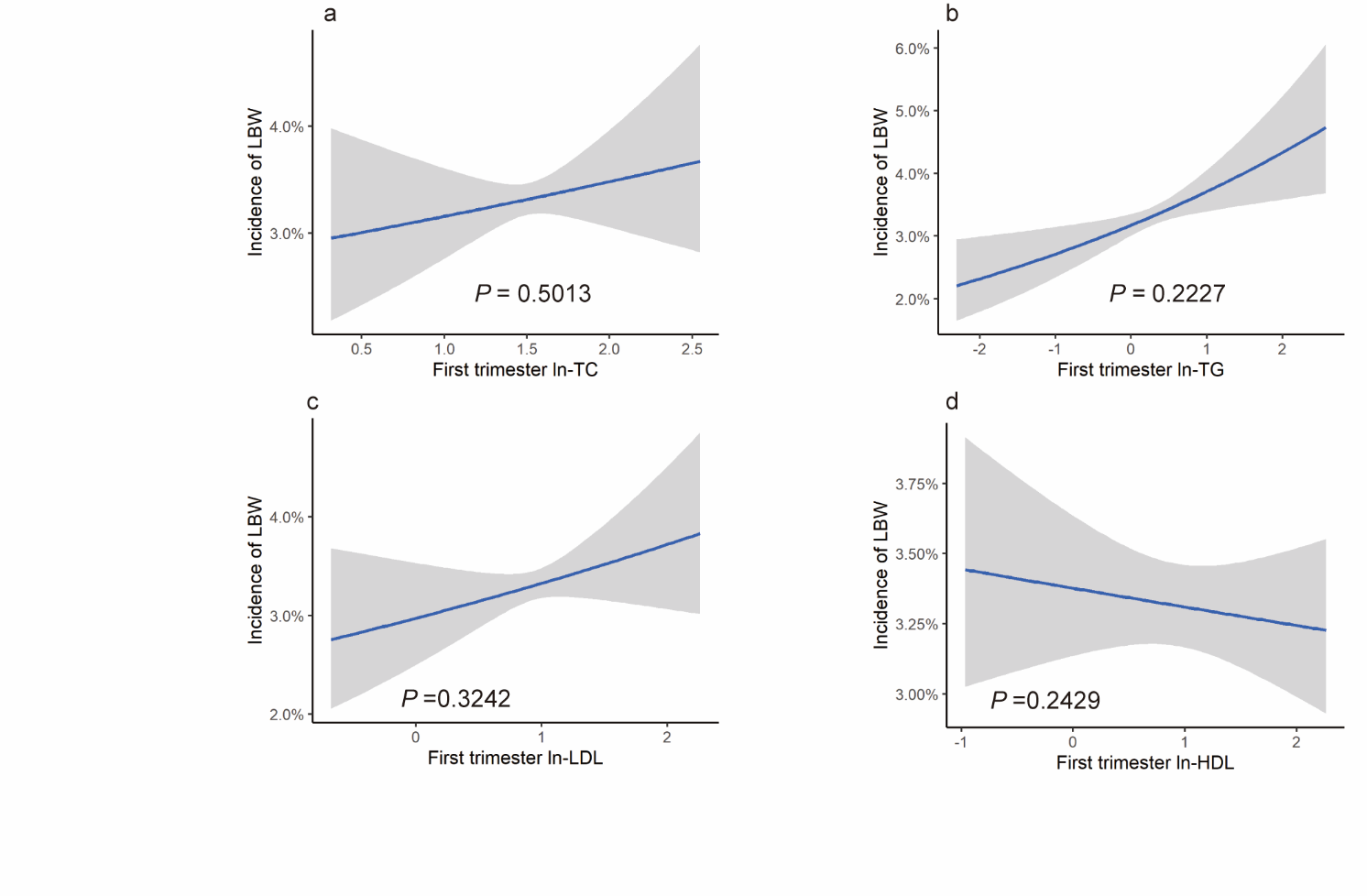


**Supplementary Figure 3.** **Risk of LBW associated with maternal lipid profiles in early pregnancy.** Logistic regression models for ln transformed (a) TC, (b) TG, (c) LDL-c, (d) HDL-c and LBW, expressed as predicted mean with 95% CIs. Analyses were adjusted for maternal pre-pregnancy BMI, age, mode of conception, parity, education, consumption of cigarettes, infant sex, gestational diabetes and gestational hypertension disorders.


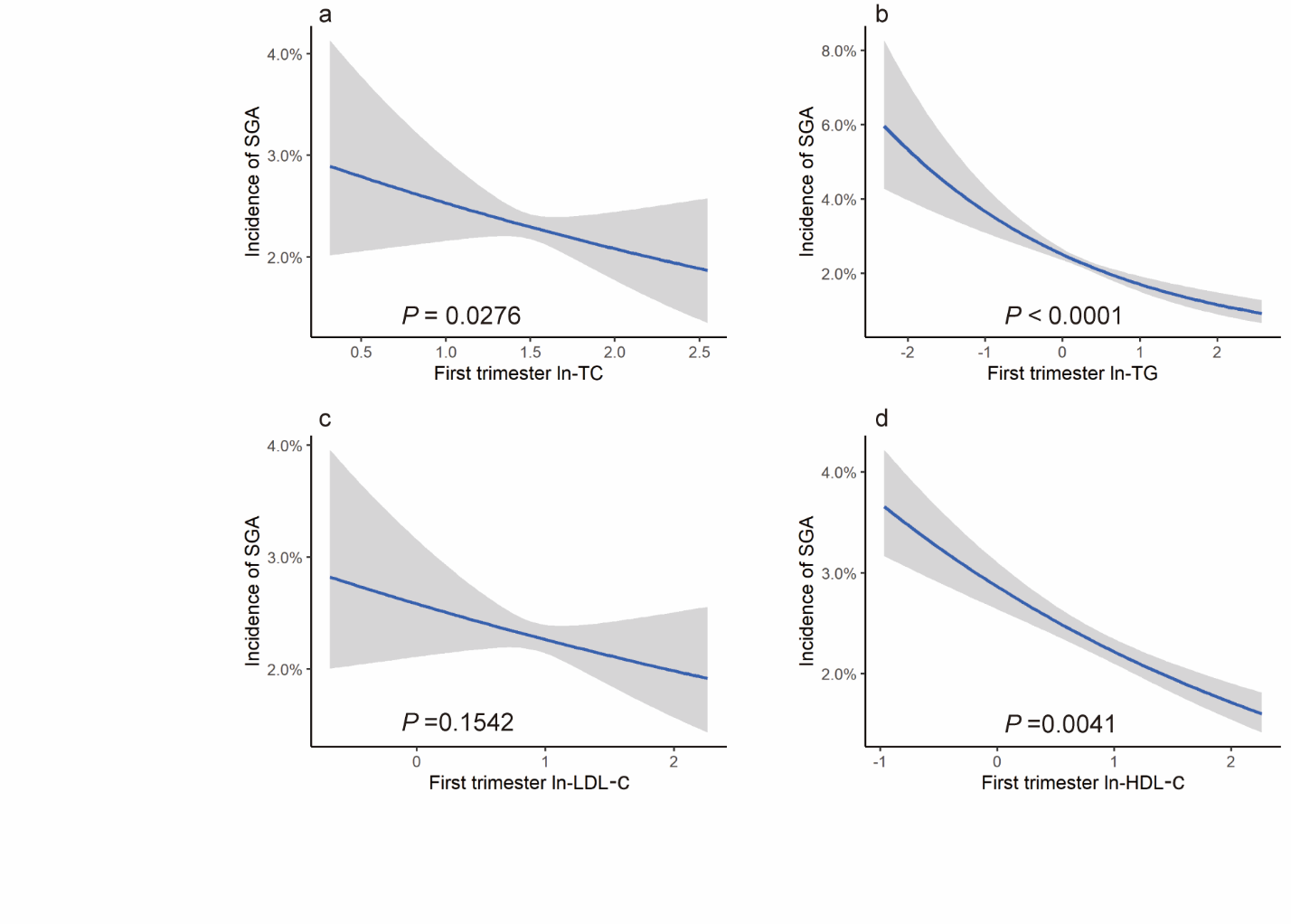


**Supplementary Figure 4. Risk of SGA associated with maternal lipid profiles in early pregnancy.** Logistic regression models for ln transformed (a) TC, (b) TG, (c) LDL-c, (d) HDL-c and SGA, expressed as predicted mean with 95% CIs. Analyses were adjusted for maternal pre-pregnancy BMI, age, mode of conception, parity, education, consumption of cigarettes, infant sex, gestational diabetes and gestational hypertension disorders.


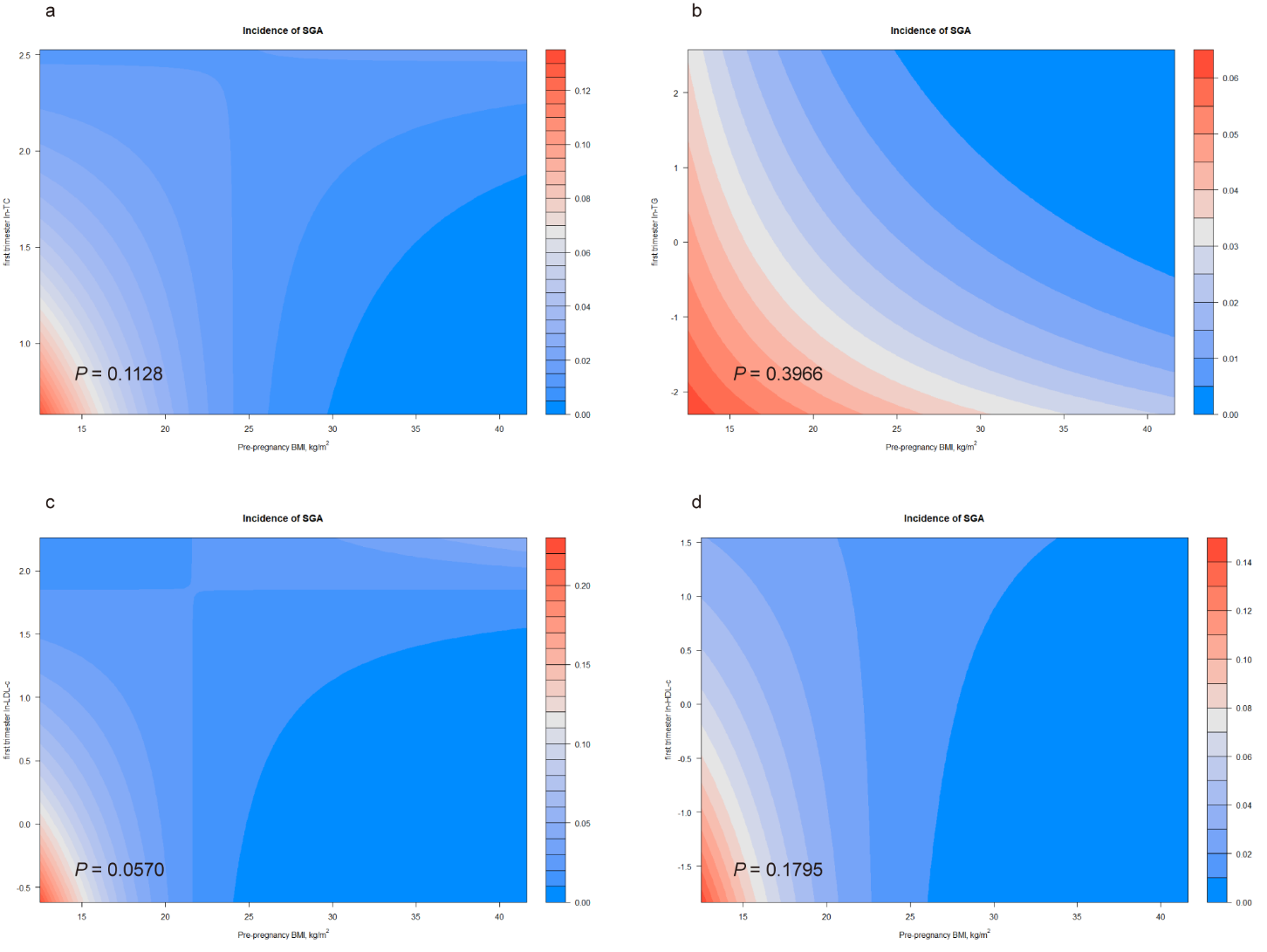


**Supplementary Figure 5.** **Combined effects of maternal pre-pregnancy BMI and lipid profiles in early pregnancy on incidence of SGA.** Heat map for the correlation of incidence of SGA (red represents increased risks of SGA, blue represents decreased risks of SGA) according to the interaction of pre-pregnancy BMI and (a) ln-TC, (b) ln-TG, (c) ln-LDL-c or (d) ln-HDL-c. Analyses were adjusted for maternal pre-pregnancy BMI, age, mode of conception, parity, education, consumption of cigarettes, infant sex, gestational diabetes and gestational hypertension disorders.


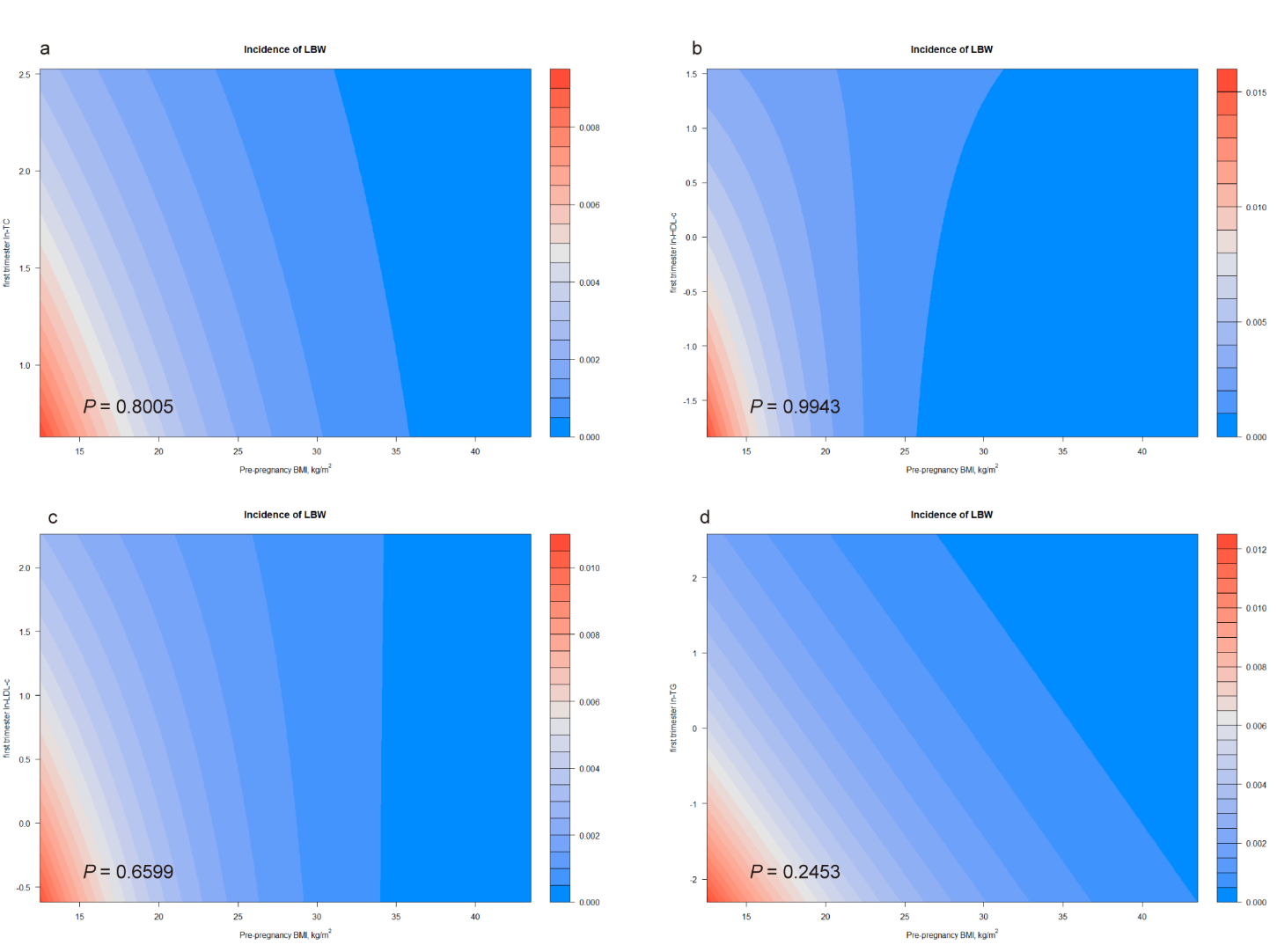


**Supplementary Figure 6.** **Combined effects of maternal pre-pregnancy BMI and lipid profiles in early pregnancy on incidence of LBW.** Heat map for the correlation of incidence of LBW (red represents increased risks of LBW, blue represents decreased risks of LBW) according to the interaction of pre-pregnancy BMI and (a) ln-TC, (b) ln-TG, (c) ln-LDL-c or (d) ln-HDL-c. Analyses were adjusted for maternal pre-pregnancy BMI, age, mode of conception, parity, education, consumption of cigarettes, infant sex, gestational diabetes and gestational hypertension disorders.


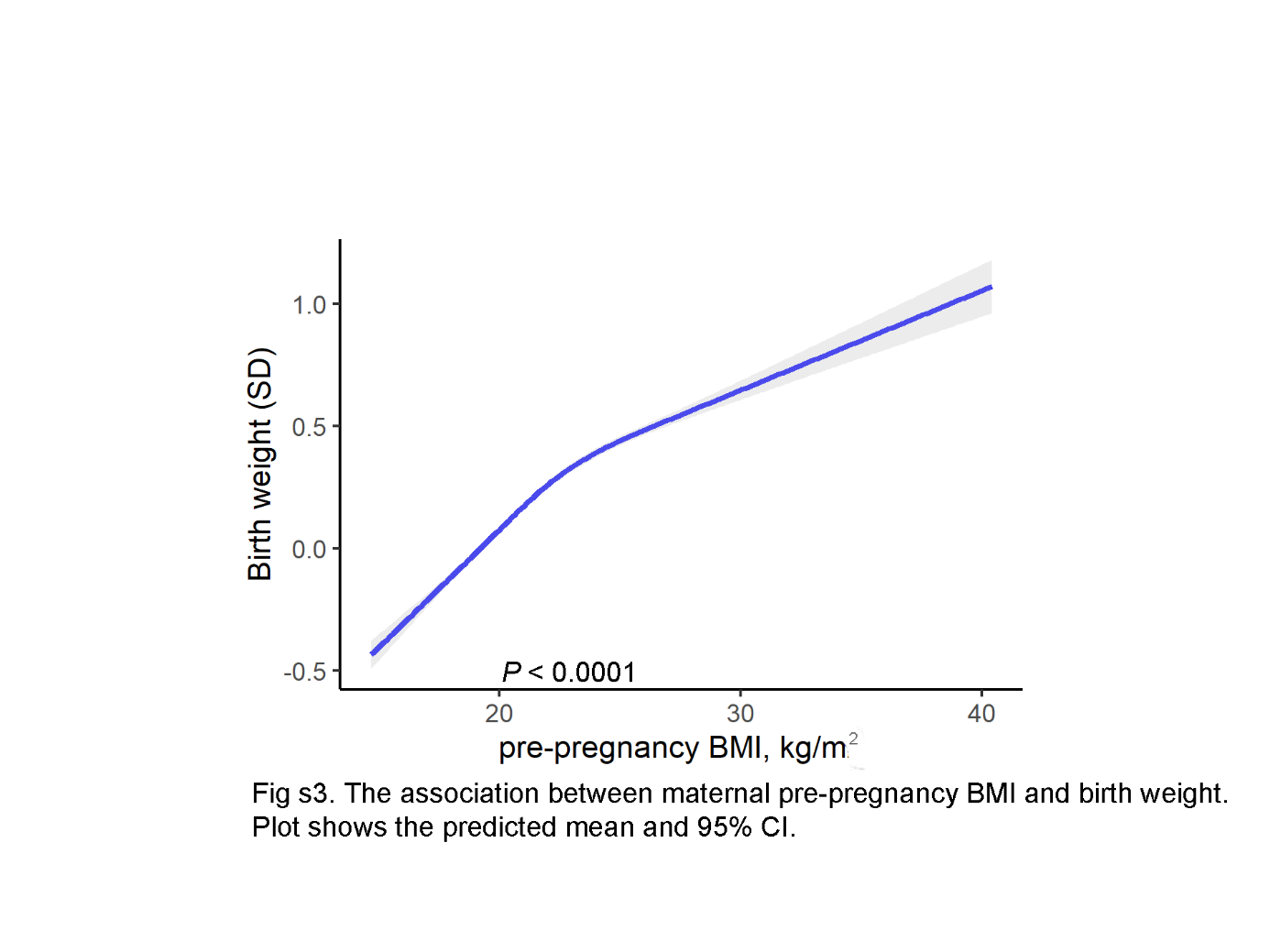


**Supplementary Figure 3. The association between maternal pre-pregnancy BMI and birth weight.** Plot shows the predicted mean and 95% CI.

| Table s1. Odds ratio of LBW, Macrosomia, SGA and LGA with per-unit increase of blood metabolic markers (ln transformed) by BMI groups. | | | | |  |
| --- | --- | --- | --- | --- | --- |
|  | **LBW** | **Macrosomia** | **SGA** | **LGA** |  |
| Normal |  |  |  |  |  |
| TC | 0.84 (0.59, 1.19) | 1.1 (0.84, 1.45) | 0.67 (0.44, 1.03) | 1.1 (0.94, 1.29) |  |
| TG | 1.11 (0.96, 1.30) | 1.33 (1.19, 1.5)* | 0.75 (0.62, 0.9)* | 1.27 (1.18, 1.36)* |  |
| LDL-c | 0.84 (0.6, 1.18) | 1.14 (0.95, 1.38) | 0.89 (0.67, 1.19) | 1.16 (1.04, 1.29)* |  |
| HDL-c | 0.91 (0.76, 1.09) | 1.04 (0.95, 1.14) | 0.82 (0.7, 0.95)* | 0.96 (0.9, 1.01) |  |
| Underweight | |  |  |  |  |
| TC | 0.97 (0.46, 2.02) | 1.91 (0.71, 5.17) | 0.48 (0.23, 1.02) | 1.04 (0.66, 1.65) |  |
| TG | 1.16 (0.82, 1.66) | 2.01 (1.27, 3.19)* | 0.69 (0.48, 0.99)* | 1.31 (1.05, 1.63)* |  |
| LDL-c | 0.77 (0.47, 1.24) | 2.03 (1.02, 4.03) | 0.56 (0.34, 0.91) | 1.37 (1.01, 1.87)* |  |
| HDL-c | 0.88 (0.62, 1.26) | 0.97 (0.68, 1.37) | 0.76 (0.59, 0.99)* | 0.77 (0.65, 0.9)* |  |
| Overweight | |  |  |  |  |
| TC | 1.25 (0.52, 2.98) | 0.84 (0.48, 1.49) | 2.6 (0.6, 11.26) | 0.89 (0.61, 1.31) |  |
| TG | 0.89 (0.62, 1.28) | 1.25 (0.98, 1.58) | 0.33 (0.18, 0.62)* | 1.24 (1.05, 1.45)* |  |
| LDL-c | 0.55 (0.22, 1.34) | 0.83 (0.57, 1.22) | 2.76 (0.93, 8.22) | 0.84 (0.65, 1.08) |  |
| HDL-c | 1.29 (0.78, 2.14) | 1.16 (0.96, 1.4) | 1.34 (0.75, 2.37) | 1.23 (1.08, 1.40)* |  |
| Obesity |  |  |  |  |  |
| TC | 2.12 (0.26, 17.3) | 1.8 (0.42, 7.76) | 1.03 (0.16, 6.79) | 1.73 (0.62, 4.82) |  |
| TG | 1.47 (0.48, 4.52) | 1.57 (0.85, 2.87) | 0.29 (0.11, 0.47)* | 1.72 (1.11, 2.65)* |  |
| LDL-c | 2.77 (0.52, 14.89) | 1.28 (0.47, 3.49) | 1.82 (0.39, 8.49) | 0.84 (0.65, 1.08) |  |
| HDL-c | 1.94 (0.4, 9.55) | 1.35 (0.83, 2.21) | 1.30 (0.49, 3.43) | 0.93 (0.66, 1.31) |  |
| a Adjusted for maternal age, mode of conception, parity, education attainment, consumption of cigarettes, infant sex, gestational diabetes and gestational hypertension disorders.  Abbreviations: LGA, large for gestational age; TC, total cholesterol; TG, triglyceride; LDL-c, low density lipoprotein cholesterol; HDL-c, high density lipoprotein cholesterol; OR, odds ratio; CI, confidential interval. | | | | |  |
|  |  |  |  |  |  |

| Table s2. Maternal lipid profiles in different pre-pregnancy categories. | | | | | |
| --- | --- | --- | --- | --- | --- |
|  | **Underweight** | **Normal** | **Overweight** | **Obesity** | ***P*** |
|  | **(n = 8024)** | **(n = 43621)** | **(n = 5153)** | **(n = 718)** |  |
| Triglyceride, Mean ± SD, mmol/L | 1.26 (0.50) | 1.41 (0.64) | 1.67 (0.77) | 1.75 (0.76) | <0.001 |
| Total cholesterol, Mean ± SD, mmol/L | 4.49 (0.83) | 4.57 (0.83) | 4.65 (0.83) | 4.69 (0.77) | <0.001 |
| Low-density lipoprotein, Mean ± SD, mmol/L | 2.49 (0.61) | 2.61 (0.63) | 2.78 (0.66) | 2.87 (0.61) | <0.001 |
| High-density lipoprotein, Mean ± SD, mmol/L | 1.71 (0.56) | 1.65 (0.52) | 1.53 (0.44) | 1.46 (0.43) | <0.001 |

| Table s3. Maternal lipid profiles and risks of LGA, macrosomia, SGA and LBW by BMI groups. | | | | | | | |
| --- | --- | --- | --- | --- | --- | --- | --- |
| percentile groups | | **LGA** | **Macrosomia** | | **SGA** | | **LBW** |
| **TC** | |  |  | |  | |  |
| <25 | | 1.02 (0.96, 1.09) | 1.08 (0.96, 1.22) | | 1.03 (0.88, 1.2) | | 1.01 (0.88, 1.15) |
| 25-50 | | 1.00 (reference) | 1.00 (reference) | | 1.00 (reference) | | 1.00 (reference) |
| 50-75 | | 1.02 (0.95, 1.08) | 1.09 (0.98, 1.21) | | 0.93 (0.79, 1.09) | | 0.98 (0.85, 1.12) |
| 75-95 | | 1.07 (0.99, 1.14) | 1.11 (0.99, 1.25) | | 0.88 (0.73, 1.06) | | 1.04 (0.89, 1.2) |
| >95 | | 1.01 (0.87, 1.17) | 1.09 (0.86, 1.39) | | 0.87 (0.59, 1.29) | | 1.03 (0.76, 1.39) |
| **TG** | |  |  | |  | |  |
| <25 | | 0.91 (0.85, 0.98) | 0.85 (0.76, 0.95) | | 1.14 (0.98, 1.33) | | 1.01 (0.88, 1.16) |
| 25-50 | | 1.00 (reference) | 1.00 (reference) | | 1.00 (reference) | | 1.00 (reference) |
| 50-75 | | 1.1 (1.03, 1.17) | 1.13 (1.02, 1.26) | | 0.85 (0.72, 1) | | 1.01 (0.88, 1.16) |
| 75-95 | | 1.19 (1.11, 1.27) | 1.14 (1.02, 1.28) | | 0.9 (0.75, 1.09) | | 1.13 (0.98, 1.31) |
| >95 | | 1.34 (1.19, 1.51) | 1.44 (1.2, 1.73) | | 0.69 (0.46, 1.04) | | 1.00 (0.77, 1.3) |
| **LDL-c** | |  |  | |  | |  |
| <25 | | 0.95 (0.88, 1.03) | 0.98 (0.86, 1.12) | | 0.93 (0.77, 1.12) | | 1.04 (0.88, 1.22) |
| 25-50 | | 1.00 (reference) | 1.00 (reference) | | 1.00 (reference) | | 1.00 (reference) |
| 50-75 | | 0.97 (0.9, 1.05) | 1.03 (0.91, 1.17) | | 0.86 (0.72, 1.04) | | 0.92 (0.78, 1.08) |
| 75-95 | | 1.04 (0.96, 1.13) | 1.18 (1.04, 1.34) | | 1.01 (0.84, 1.23) | | 1.05 (0.89, 1.24) |
| >95 | | 1.01 (0.88, 1.16) | 1.07 (0.86, 1.34) | | 0.77 (0.52, 1.13) | | 0.96 (0.72, 1.29) |
| **HDL-c** | |  |  | |  | |  |
| >75 | | 0.87 (0.8, 0.94) | 0.81 (0.71, 0.94) | | 0.96 (0.78, 1.18) | | 0.88 (0.74, 1.04) |
| 50-75 | | 1.00 (reference) | 1.00 (reference) | | 1.00 (reference) | | 1.00 (reference) |
| 25-50 | | 1.03 (0.95, 1.12) | 1.02 (0.89, 1.16) | | 0.96 (0.78, 1.19) | | 0.9 (0.76, 1.06) |
| 5-25 | | 0.99 (0.91, 1.09) | 1.00 (0.87, 1.15) | | 0.99 (0.8, 1.23) | | 0.78 (0.65, 0.94) |
| >95 | | 1.02 (0.90, 1.16) | 0.98 (0.80, 1.20) | | 1.22 (0.92, 1.61) | | 0.75 (0.57, 0.98) |
| Data are shown as OR and 95% CI, adjusted for maternal age, mode of conception, parity, education attainment, consumption of cigarettes, infant sex, gestational diabetes and gestational hypertension disorders. LGA, large for gestational age; LBW, low birth weight; SGA, small for gestational age; TC, total cholesterol; TG, triglyceride; LDL-c, low density lipoprotein cholesterol; HDL-c, high density lipoprotein cholesterol. | | | | | | | |
| Table s4. Sensitivity analysis between matenal lipid profiles and birth weight (SD) after excluding GDM and gestational hypertention disorders population. | | | | | | | |
|  | β (95% CI) | | | *P* value | |  |  |
| ln-TC | 0.090 (0.037, 0.146) | | | 0.0011 | |  |  |
| ln-TG | 0.155 (0.131, 0.179) | | | <0.0001 | |  |  |
| ln-LDL-c | 0.072 (0.045, 0.118) | | | <0.0001 | |  |  |
| ln-HDL-c | 0.026 (-0.087, 0.056) | | | 0.6317 | |  |  |
| Abbreviations: TC, total cholesterol; TG, triglyceride; LDL-c, low density lipoprotein cholesterol; HDL-c, high density lipoprotein cholesterol. | | | | | |  |  |
